# HMGA1 Induction of miR-103/107 Forms a Negative Feedback Loop to Regulate Autophagy in MPTP Model of Parkinson’s Disease

**DOI:** 10.3389/fncel.2020.620020

**Published:** 2021-01-18

**Authors:** Gehui Li, Wanxian Luo, Baoyan Wang, Chen Qian, Yongyi Ye, Yuantao Li, Shizhong Zhang

**Affiliations:** ^1^Guangdong Provincial Key Laboratory on Brain Function Repair and Regeneration, The National Key Clinical Specialty, Department of Neurosurgery, The Engineering Technology Research Center of Education Ministry of China, The Neurosurgery Institute of Guangdong Province, Zhujiang Hospital, Southern Medical University, Guangzhou, China; ^2^Department of Anesthesiology, Shenzhen Maternity and Child Healthcare Hospital, Southern Medical University, Shenzhen, China; ^3^Department of Medicine Ultrasonics, Nanfang Hospital, Southern Medical University, Guangzhou, China; ^4^Department of Neurosurgery, The Second Affiliated Hospital of Guangzhou Medical University, Guangzhou, China

**Keywords:** Parkinson’s disease, dopaminergic neuron, autophagy, HMGA1, miR-103/107

## Abstract

Autophagy dysfunction has been directly linked with the onset and progression of Parkinson’s disease (PD), but the underlying mechanisms are not well understood. High-mobility group A1 (HMGA1), well-known chromatin remodeling proteins, play pivotal roles in diverse biological processes and diseases. Their function in neural cell death in PD, however, have not yet been fully elucidated. Here, we report that HMGA1 is highly induced during dopaminergic cell death *in vitro* and mice models of PD *in vivo*. Functional studies using genetic knockdown of endogenous HMGA1 show that HMGA1 signaling inhibition accelerates neural cell death, at least partially through aggravating MPP^+^-induced autophagic flux reduction resulting from partial block in autophagic flux at the terminal stages, indicating a novel potential neuroprotective role for HMGA1 in dopaminergic neurons death. MicroRNA-103/107 (miR-103/107) family, which is highly expressed in neuron, coordinately ensures proper end-stage autophagy. We further illustrate that MPP^+^/1-methyl-4-phenyl-1,2,3,6-tetrahydropyridine (MPTP)-induced HMGA1 elevation counterparts the effect of miR-103/107 downregulation by directly binding to their promoters, respectively, sustaining their expression in MPP^+^-damaged MN9D cells and modulates autophagy through CDK5R1/CDK5 signaling pathway. We also find that HMGA1 is a direct target of miR-103/107 family. Thus, our results suggest that HMGA1 forms a negative feedback loop with miR-103/107-CDK5R1/CDK5 signaling to regulate the MPP^+^/MPTP-induced autophagy impairment and neural cell death. Collectively, we identify a paradigm for compensatory neuroprotective HMGA1 signaling in dopaminergic neurons that could have important therapeutic implications for PD.

## Introduction

The high-mobility group A1 (HMGA1) proteins are members of the high mobility group (HMG) superfamily, which mainly locate in the nucleus and function as chromatin remodeling factors (Resar, 2010; Schuldenfrei et al., 2011). Prior studies reveal that HMGA1 proteins regulate the transcription of genes primarily through binding to specific DNA sequences (AT-rich regions in the minor groove), altering chromatin structure, and recruiting other transcription factors (D’Angelo et al., 2017; Sgarra et al., 2018). They are considered to serve as critical genetic regulators and involve in diverse physiological and pathological progresses, including cell cycle regulation (Fedele et al., 2005), differentiation (Giannini et al., 2000), apoptosis (Pierantoni et al., 2007), cellular metabolism (Ha et al., 2012), mitochondrial regulation (Dement et al., 2007; Mao et al., 2009), and autophagic signaling pathway (Conte et al., 2017; Wu et al., 2020). Indeed, a large amount of evidence has demonstrated the pivotal role of HMGA1 in diverse, aggressive cancers and normal development (Schoenmakers et al., 1995; Wood et al., 2000; Reeves, 2001). Moreover, recent studies have also shown a relationship between aberrant high expression of HMGA1 and Alzheimer’s disease, a famous neurodegenerative disorder (Sumter et al., 2016). It has been shown that HMGA1a functioned as a pre-mRNA binding protein inducing aberrant alternative splicing of PS2 pre-mRNA (Ohe et al., 2018), indicating its implication in neurodegenerative conditions. However, its role in Parkinson’s disease (PD), another most common movement disorder, has remained elusive.

Since the first description of PD nearly two centuries ago, tens of thousands of studies have been conducted to dissect the underlying molecular mechanisms of dopaminergic neuron loss in the substantia nigra (Surmeier et al., 2017; Ye et al., 2018; Van Bulck et al., 2019). Autophagy, a major lysosomal-dependent protein degradation process used to eliminate damaged organelles and protein aggregates, is now receiving particular attention, as compelling evidences show that dysfunctional autophagy might contribute to the cascade of events leading to neuronal death (Nixon, 2013; Wang et al., 2016; Menzies et al., 2017). Indeed, autophagy is defective at multiple stages in Parkinson’s disease, such as autophagy induction, cargo sequestration, and lysosomal digestion of substrates (Nixon, 2013). Increasing autophagosome formation is found in postmortem PD brains (Parekh et al., 2019), 1-methyl-4-phenyl-1,2,3,6-tetrahydropyridine (MPTP) and A53T α-synuclein mutant mouse model of PD (Jiang and Dickson, 2018). Neurotoxin MPTP treatment also induced impaired autophagosome degradation resulting from impaired lysosomal activity (Lim et al., 2011; Miyara et al., 2016). Importantly, based on current evidence, autophagy modulation seems to be an effective interventional strategy for most of the neurodegenerative disorders. Thus, uncovering the critical autophagic events underlying PD is of great importance.

Here, by using series of *in vitro* and *in vivo* experiments, we demonstrated autophagy regulation as a mechanism by which HMGA1 contributes to neuronal loss in PD models. We found that HMGA1 was highly induced during dopaminergic neural cell death triggered by MPTP/MPP^+^ (1-methyl-4-phenylpyridinium, active metabolite of MPTP) treatment. Functional studies using genetic knockdown of endogenous HMGA1 showed that HMGA1 signaling inhibition accelerated neural cell death through promoting MPP^+^-induced autophagy impairment, indicating a novel potential neuroprotective role for HMGA1 in dopaminergic cells during neuronal death. Given that HMGA1 is involved in regulation of microRNAs (miRNAs) expressions, we further illustrated that HMGA1 regulated the expression of miR-103/107 family by directly binding to their promoters, respectively, and modulated CDK5R1/CDK5 signaling pathway. More importantly, we surprisingly found that HMGA1 was also a direct target of miR-103/107 family. Thus, in this study, we proposed a negative feedback loop between HMGA1 and miR-103/107 family and depicted its pivotal role during autophagy impairment and neural cell death in PD.

## Materials and Methods

### Cell Culture and Treatment

MN9D cells were maintained in Dulbecco’s modified Eagle’s medium (DMEM; Invitrogen, Carlsbad, CA, USA) supplemented with 10% fetal bovine serum (Gibco, Grand Island, NY, USA), penicillin (100 IU/ml), and streptomycin (100 mg/ml) at 37°C in a humidified incubator with 5% CO_2_. For cell treatment, MN9D cells were treated with MPP^+^ (Sigma–Aldrich, St. Louis, MO, USA) at different concentrations for indicated times. Bafilomycin A1 (100 nM, Selleck Chemicals, Shanghai, China) was used in this study. Cell death assays were analyzed by flow cytometry (Becton-Dickinson Immunocytometry Systems, San Jose, CA, USA) using an Annexin V-FITC and propidium iodide (PI) kit (Invitrogen, Carlsbad, CA, USA).

### Oligonucleotide Transfection

Small-interfering RNA (siRNA) oligos targeting mouse HMGA1 (#1 GACCAAAGGGAAGCAAGAAUA, #2 AGUGAUCACCACUCGCAGUGC), mouse CDK5 (#1 UAUAAGCCCUAUCCGAUGU, #2 GGAUUCCCGUCCGCUGUUA), and negative control (NC) siRNA (UUCUCCGAACGUGUCACGUTT) were synthesized by GenePharma Biotechnology (Shanghai, China). MiR-103/107 mimics, inhibitors, and negative controls (mimic control, inhibitor control) were purchased from RiboBio (Guangzhou, China). These oligonucleotides were all transfected into MN9D cells using Lipofectamine^®^ RNAiMAX^TM^ (Invitrogen) at a final concentration of 100 nM, according to the manufacturer’s instructions.

### Adenoviral Constructions and Purification

The HMGA1 knockdown adenoviruses were constructed as previously described (Ye et al., 2018). Briefly, HMGA1 short hairpin RNA (shRNA) harboring siRNA#1 (Ad-HMGA1-shRNA) or control shRNA (Ad-control) were inserted into pDC315-U6-shRNA, respectively. Adenoviruses were then produced by cotransfecting HEK293T cells with each adenoviral construct together with the packaging vectors pBHGloxdelE13cre (Microbix Biosystems, Mississauga, ON, Canada) using Lipofectamine 2000 (Life Technologies), according to the instructions of the manufacturer. Adenoviruses were then amplified and purified using the ViraTrapTM adenovirus purification kit (Biomiga Inc., San Diego, CA, USA).

### RNA Isolation and Real-Time PCR

Total RNA was extracted from cells and mouse brain tissue samples using RNAiso Plus (Takara, Japan) according to the manufacturer’s protocol. For messenger RNA (mRNA) expression assessments, 1 μg total RNA was used to synthesize complementary DNA using a PrimeScript^TM^ RT reagent kit with gDNA Eraser (Takara, Japan). Complementary DNA (cDNA) products were then diluted 1:10 in ddH_2_O. Quantitative real-time PCR (qRT-PCR) was performed using the Roche LightCycler480 (Roche Diagnostics, Mannheim, Germany) with SYBR^®^ Premix Ex Taq^TM^ II.

The primer sequences used were as follows: mouse HMGA1, forward CAAGACCCGGGAAAGTCA, reverse CAGAGGACTCCTGGGAGATG; β-actin, forward CTAAGGCCAACCGTGAAAAG, reverse ACCAGAGGCATACAGGGACA. For microRNAs, total RNA was reverse transcribed using the mmu-miR-103 and mmu-miR-107 qRT-PCR kit (RIBOBIO, Guangdong, China) according to the manufacturer’s instructions. U6 small nuclear RNA (snRNA; for miRNAs) or β-actin expression (for mRNAs) were used as control for normalization, and the relative gene expression levels were calculated using the 2^−ΔΔCt^ method.

### Immunoblot Analysis

Total protein samples were collected from cells or selected mouse midbrain with standard radioimmunoprecipitation assay (RIPA) buffer (Beyotime, Jiangsu, China) containing protease and phosphatase inhibitor cocktails (Selleck Chemicals, Shanghai, China). Equal amounts of the proteins were loaded on sodium dodecyl sulfate–polyacrylamide gel electrophoresis (SDS–PAGE) gels for separation and transferred to polyvinylidene fluoride (PVDF) membrane (Immobilon-P, Millipore, Danvers, MA, USA) in a Mini Trans-Blot electrophoretic transfer cell (Bio-Rad). After blocking with 5% nonfat milk, the membranes were incubated with one of the following primary antibodies: anti-HMGA1 (1:1,000, Abcam, ab4078), anti-LC3 (1:5,000, Sigma–Aldrich, L7543), anti-p62 (1:5,000, Sigma–Aldrich, P0067), anti-AKT (1:1,000, Cell Signaling, #4685), anti-p-AKT (Ser473, 1:1,000, Cell Signaling, #9271), anti-mTOR (1:1,000, Cell Signaling, #2983), anti-p-mTOR (Ser2448, 1:1,000, Cell Signaling, #2971), anti-4EBP1 (1:1,000, Cell Signaling, #9644), anti-p-4EBP1 antibody (Ser65, 1:1,000, Cell Signaling, #9451), anti-CDK5 (1:1,000, Cell Signaling, #14145), anti-CDK5R1 (1:1,000, Cell Signaling, #2680), or anti-β-actin (1:1,000, Cell Signaling, #4970) overnight at 4°C. Horseradish peroxidase (HRP)-conjugated secondary antibody (goat antirabbit IgG 1:5,000) was used for antibody detection with the immobilon ECL kit (Merck Millipore). Relative intensities of each band were measured using ImageJ.

### Luciferase Reporter Assay

The sequence from wild-type or mutants 3′-untranslated region (3′-UTR) of HMGA1 containing miR-103/107 family binding site were synthesized and cloned into the pmirGLO vector (Promega, Madison, WI, USA). For mutated 3′-UTRs, seven bases of seed region were replaced with complementary bases. For the microRNA luciferase reporter assays, HEK293T cells were cotransfected with the indicated luciferase constructs with miR-103 or miR-107 mimics (Ribobio, Guangzhou, China). After 48 h, the luciferase activity was measured using the dual-luciferase reporter assay system (Promega) according to the manufacturer’s instructions.

### Autophagic Flux Analysis

The mRFP-GFP-LC3 adenoviral particles were purchased from HanBio (Shanghai, China). For the assessment of autophagic flux, MN9D cells were infected with Ad-mRFP-GFP-LC3 according to the manufacturer’s instructions. Twenty-four hours after, MN9D cells infected with Ad-mRFP-GFP-LC3 were transfected with HMGA1 siRNA or NC, followed by MPP^+^ (200 μM) or phosphate-buffered saline (PBS) treatment. LC3 puncta were examined with a Zeiss LSM 880 laser-scanning microscope fitted with Airyscan module and Plan-Apochromat 63× 1.4 oil DIC lens (Carl Zeiss, Jena, Germany). To quantify the LC3 punctate staining pattern per cell, at least 30 cells were randomly selected from each of three independent experiments and analyzed using ImageJ Imaging software.

### Chromatin Immunoprecipitation Assay

MN9D cells were processed for chromatin immunoprecipitation (ChIP) experiments as reported (Fu et al., 2018) using the EZ ChIP^TM^ Chromatin Immunoprecipitation Kit (Millipore, Danvers, MA, USA). The chromatin of MN9D cells was cross-linked and immunoprecipitated with the specific HMGA1 (Abcam, ab4078) antibody and rabbit IgG (Beyotime, A7016). The purified DNAs were analyzed by qRT-PCR using the following primer sequences: miR-103 ChIP1, forward TCACTGTGAAGCCCAGGTC, reverse TGCTAAGCATGGTGGCACAG; ChIP2, forward TCAGTGCTCTTACACTGAGCC, reverse ACACATGTGCTCTTAACACAAAAA; ChIP3, forward GACAGGGAATGCTACCTGTTCA, reverse GATGGGAGCATCCACTGAAA; miR-107 ChIP1, forward GTCCCCAGCTACACCAAGAC, reverse TTATAACGCTGGCCCCTGAC.

### Animals and Animal Model

C57BL/6 mice (male, 8–10 weeks) were purchased from the Laboratory Animal Centre of Southern Medical University. Animal care and procedures were carried out with the approval of the Southern Medical University Ethics Committee and strictly complied with the National Institutes of Health (NIH) Guide for the Care and Use of Laboratory Animals. The well-characterized subacute MPTP mouse model of PD was used. In brief, a single injection of MPTP-HCl (25 mg/kg free base) or equivalent saline was administered subcutaneously to the mice daily for five consecutive days. Mice were sacrificed at indicated time points: 0 (immediately after the last MPTP injection), 1, 2, 4, and 7 days after the last MPTP injection for either immunofluorescence, qRT-PCR, or Western blotting.

### Stereotaxic Surgery

Stereotaxic surgery was carried out with a stereotaxic frame (Stoelting, Wood Dale, IL, USA) and a 5-μl Hamilton syringe to inject the adenoviruses directly into the right substantia nigra region. The following stereotaxic coordinates were used: AP, 3.1 mm; ML, 1.2 mm; DV, 4.0 mm from Bregma. After drilling a hole in the skull, adenoviruses (2 μl; 1 × 10^10^ IFU/μl per construct) were stereotactically delivered into the brain. Four weeks later, the mice were treated with either saline or MPTP as described above. All mice were sacrificed at the indicated time points.

### Immunofluorescence and Image Analysis

For immunofluorescence study, cells were fixed with 4% paraformaldehyde (PFA) at room temperature (RT) for 10 min. Slides were incubated overnight with antibodies against HMGA1 (1:500, Abcam, ab4078). After washing, slides were incubated with goat antirabbit IgG conjugated to Alexa Fluor 488 (1:400, Servicebio, Wuhan, China). 4,6-Diamidino-2-phenylindole (DAPI, 1:1,000, Roche) was used to identify nuclei. For histology, animals were anesthetized and transcardially perfused with PBS followed by ice-cold 4% paraformaldehyde. Brains were postfixed in the same fixative for 2 days at 4°C and then cryoprotected in 30% sucrose for another 2 days at 4°C. A series of mesencephalic coronal sections (12 μm) were collected using a freezing microtome (Leica) and mounted on poly-D-lysine (PDL)-coated slides. Rabbit anti-HMGA1 (1:500, Abcam, ab4078) or mouse anti-TH (1:250, Millipore, Danvers, MA, USA) antibodies were incubated with the sections overnight at 4°C, followed by incubation with goat antirabbit IgG conjugated to Alexa Fluor 488 (1:400, Servicebio, China) or Cy3-conjugated goat antimouse IgG (1:300, Servicebio, Wuhan, China), respectively. DAPI was used to identify nuclei. For each animal, total numbers of TH+ neurons in the substantia nigra region were manually counted as previously described (Zhang et al., 2004; Wang et al., 2015). Briefly, TH+ cell counting was performed under an Olympus DP70 microscope (200×; Olympus America Inc., Melville, NY, USA). The boundary between the SNpc and the adjacent ventral tagmental area was defined following the mouse brain atlas (Nelson et al., 1996). The total number of immunoreactive cells in the entire extent of the SNpc was counted from four mouse brains per group. Each brain contained eight sections/brain, which was performed by three to four individuals who were blind to the treatment. A mean value for the number of SNpc TH+ neurons was then deduced by averaging the counts of the eight sections for each animal; the results were expressed as the average number of TH+ neurons per SNpc.

### Statistical Analysis

The data are shown as means ± standard error (SEM). Statistical significance was determined by two-tailed unpaired Student’s *t*-test or analysis of variance (ANOVA) with Bonferroni *post hoc* tests. *P* < 0.05 was considered statistically significant.

## Results

### HMGA1 Is Upregulated During MPP^+^-Induced Neuronal Cell Death

**Figure 1:**
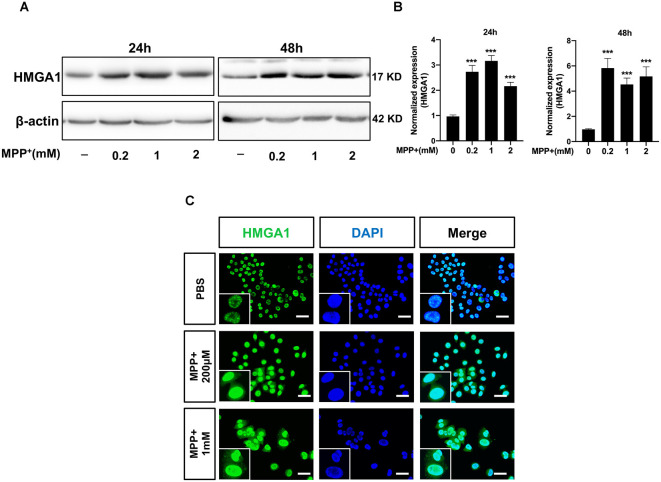
High-mobility group A1 (HMGA1) is upregulated during MPP^+^-induced neuronal cell death. **(A,B)** MN9D cells were treated with different concentrations of MPP^+^ for 24 or 48 h, and expression levels of HMGA1 were analyzed by Western blotting. **(C)** Localization of HMGA1 (green) in MN9D cells was determined by immunofluorescence after exposure to MPP^+^ for 24 h. 4′,6-Diamidino-2-phenylindole (DAPI) was used to stain the nuclei (blue). Scale bar: 50 μm. Data are expressed as mean ± SEM (*n* = 3). *P < 0.05, **P < 0.01, ***P < 0.001 vs. control using one-way analysis of variance (ANOVA) followed by Bonferroni test.

To explore the role of HMGA1 in PD, we first measured the expression levels of HMGA1 in Parkinsonian toxicant MPP^+^-treated MN9D cells (mesencephalon-derived dopaminergic neuronal cell line). As shown in Figures 1A,B, the HMGA1 protein was significantly upregulated at both 24 and 48 h following treatment with different concentrations of MPP^+^ (0.2, 1, and 2 mM). To further analyze the expression level and localization of HMGA1, immunofluorescence detection was also performed. As shown in Figure 1C, HMGA1 was elevated and showed a primary nuclear localization pattern in MN9D cells. Thus, these results identify HMGA1 as an inducible mediator during neurotoxic stress.

### HMGA1 Is Induced in Nigral DA Neurons in MPTP-Treated Mice

**Figure 2:**
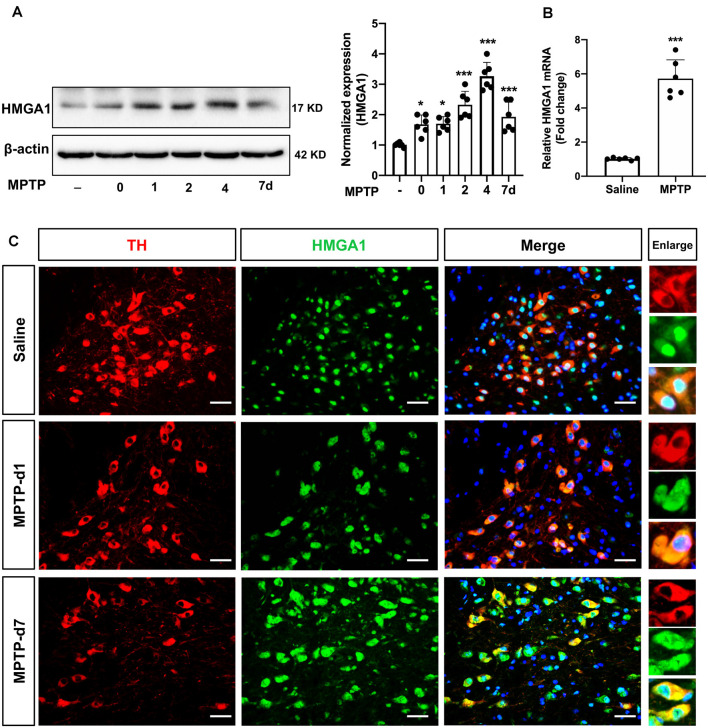
High-mobility group A1 (HMGA1) is highly induced in nigral DA neurons in 1-methyl-4-phenyl-1,2,3,6-tetrahydropyridine (MPTP) mouse model of Parkinson’s disease (PD). **(A,B)** Mice received five daily injections of MPTP (25 mg/kg/day intraperitoneally) or saline and were sacrificed at indicated time. Total protein and RNA isolated from dissected ventral mesencephalon were subjected to **(A)** Western blot and **(B)** qRT-PCR analysis of HMGA1. Data are expressed as mean ± SEM (*n* = 6 mice per group). *P < 0.05, ***P < 0.001 vs. control group using one-way analysis of variance followed by Bonferroni test or two-tailed unpaired Student’s *t-test*. **(C)** Representative immunofluorescence double staining for tyrosine hydroxylase (TH; red) and HMGA1 (green) showing increased HMGA1 expression in nigral dopaminergic neurons. Scale bar: 50 μm.

We next determined HMGA1 expression in the substantia nigra (SN) region *in vivo*. The well-characterized subacute MPTP mouse model of PD was used, that is, five daily injections of MPTP (25 mg/kg/day intraperitoneally). Western blot and densitometric analysis (Figure 2A) revealed that, in concordance with our *in vitro* results, MPTP treatment greatly induced the expression of HMGA1 in nigral tissue lysates at different timepoints after the final MPTP injection. qRT-PCR analysis also confirmed the upregulation of HMGA1 mRNA (Figure 2B). Moreover, we further identified that, in MPTP-treated mice, intense HMGA1 staining was largely localized with surviving tyrosine hydroxylase positive (TH+) dopaminergic neurons, and HMGA1 elevation was observed both in the cell nucleus and cytoplasm, while in the saline-treated group, HMGA1 was mainly expressed in cell nucleus with low expression level (Figure 2C, Supplementary Figure 1). Notably, as shown in Figure 2C, elevated HMGA1 was also found in other cell types (TH-negative) both in saline/MPTP-treated mice, indicating its multiple roles in regulating cell functions. Indeed, Rayaprolu et al. (2020) recently reported that HMGA1 was most highly expressed at the protein level in the microglia. Together, these results demonstrate for the first time that HMGA1 expression is elevated in subacute MPTP mouse model of PD.

### Knockdown HMGA1 Exacerbates Dopaminergic Cell Death

**Figure 3:**
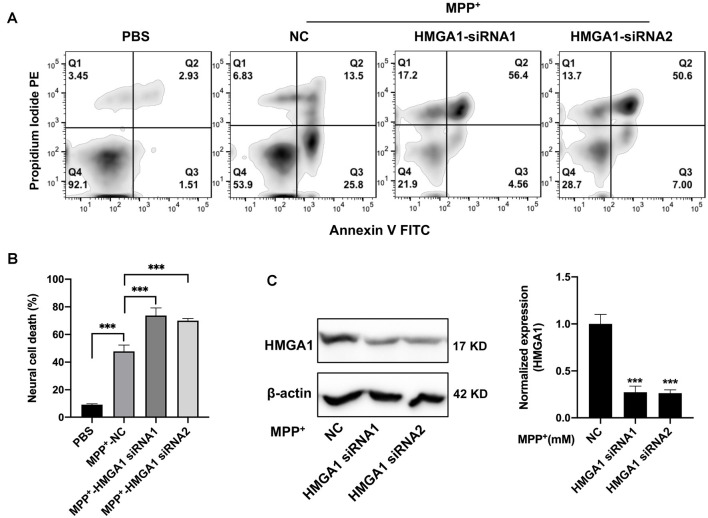
Loss of high-mobility group A1 (HMGA1) exacerbates dopaminergic cell death. **(A,C)** MN9D cells were transfected with two HMGA1 small-interfering RNAs (siRNAs) or negative control (NC), respectively, and exposed to MPP^+^ (200 μM) for 48 h. Representative images of MN9D cell deaths assessed by flow cytometry **(A)** analysis and **(B)** quantification were shown. **(C)** MN9D cells were transfected with HMGA1 siRNA or NC, and HMGA1 levels were analyzed by Western blot. ***P < 0.001 vs. control using one-way ANOVA followed by Bonferroni test.

We then examined the physiological roles of HMGA1 during dopaminergic cell death. Prior studies have been reported that HMGA1 promotes an antiapoptotic response (Pierantoni et al., 2007). We than used two siRNAs targeting HMGA1 to specifically suppress its level in MN9D cells and studied the dopaminergic cell death under MPP^+^ treatment. Successful suppression of HMGA1 protein was confirmed by Western blotting (Figure 3C). Twenty-four hours after transfection with HMGA1 siRNA or NC, MN9D cells were treated with 200 μM MPP^+^ for another 48 h and resorted to flow cytometry analysis. Not surprisingly, MPP^+^ exposure led to significant cell death, while HMGA1 knockdown further exacerbated MPP^+^-mediated cell death, indicating that HMGA1 may be protective in dopaminergic cells during neuronal death (Figures 3A,B). In summary, our data demonstrate that HMGA1 induction is critical to protect dopaminergic cells against MPP^+^-induced degeneration.

### HMGA1 Depletion Promotes MPP^+^-Induced Autophagy Impairment

**Figure 4:**
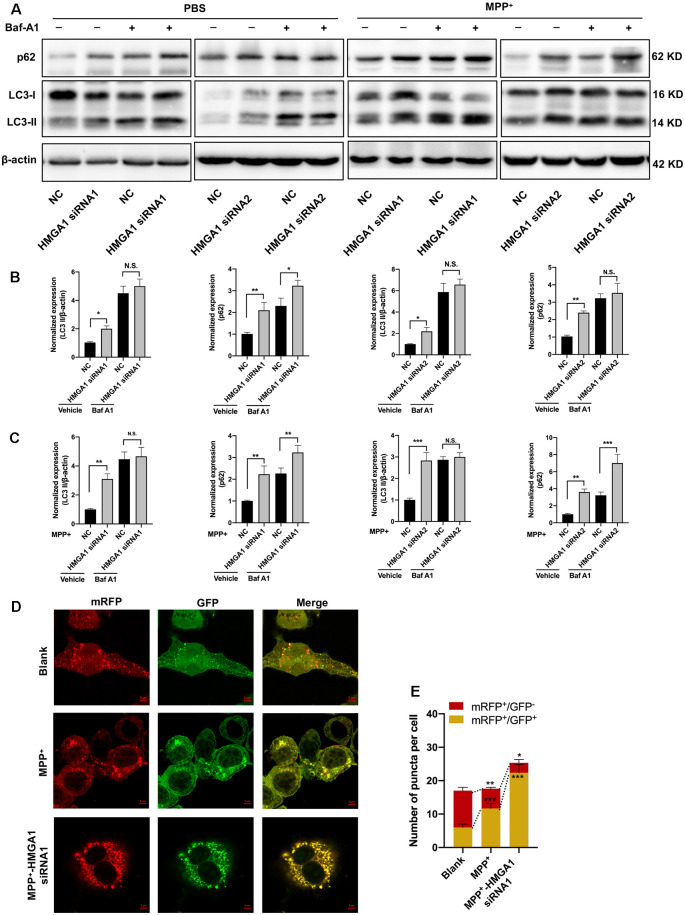
High-mobility group A1 (HMGA1) depletion promotes MPP^+^-mediated autophagy impairment. **(A–C)** MN9D cells were transfected with two HMGA1 small-interfering RNAs (siRNAs) or NC, respectively, and exposed to MPP^+^ (200 μM) for 48 h in the presence or absence of lysosomal pump inhibitor Bafilomycin A1 Baf-A1; (100 nM) for the last 4 h. **(A)** Immunoblot analysis of LC3 and p62 levels and **(B,C)** quantification were shown. **(D)** MN9D cells infected with Ad-mRFP-GFP-LC3 were transfected with HMGA1 siRNA or NC, followed by MPP^+^ (200 μM) or phosphate-buffered saline (PBS) treatment.Representative confocal images are shown. Scale bar, 5 μm. **(E)** Quantification of the number of puncta per cell that were mRFP^+^/GFP^+^ or mRFP^+^/GFP^−^ were shown. Data are expressed as mean ± SEM (*n* = 3). N.S., not statistically significant. *P < 0.05, **P < 0.01, and ***P < 0.001 vs. control using one-way or two-way ANOVA followed by Bonferroni test.

Having found that HMGA1 depletion is neurotoxicity, we next investigated the mechanisms underlying this effect. A connection between HMGA1 and autophagy regulation has already been reported by Conte et al. (2017). Given the emerging link between autophagic flux deregulation and neuronal loss triggered by MPTP/MPP^+^ toxicity, it is plausible that the effect of HMGA1 on autophagy contributes to the pathophysiology of PD. We thus examined whether HMGA1 takes part in the regulation of autophagy in MN9D cells. During autophagosomes formation, LC3-I converses to LC3-II, a common hallmark of autophagy, by being conjugated to the membrane lipid phosphatidylethanolamine (He and Klionsky, 2009). As shown in Figures 4A,B, HMGA1 knockdown alone induced the accumulation of endogenous LC3-II. This can result from either enhanced autophagosome formation or impaired degradation or both. We thus further performed LC3-II turnover assay (Sarkar et al., 2009), with the use of lysosomal vacuolar-type H^+^-ATPase (V-ATPase) pump inhibitor Bafilomycin-A1 (Baf-A1), to inhibit autophagosome fusion with lysosomes and subsequent degradation by preventing autophagosome acidification and maturation. As shown in Figure 4A, the knockdown of HMGA1 plus Baf-A1 did not further increase LC3-II levels compared to Baf-A1 alone, indicating that the knockdown of HMGA1 induced a block in autophagic flux at the terminal stages. While the knockdown of HMGA1 plus baf-A1 caused a massive increase in LC3-II compared with HMGA1 knockdown alone, this indicated that the autophagic flux at the terminal stages was not completely blocked. The increased expression of p62, a well-known autophagic cargo protein constantly degraded by the autophagy-lysosome system (Klionsky et al., 2016), further supported the reduction in autophagic flux in HMGA1-knockdown MN9D cells (Figures 4A,B). We then identified the potential roles of HMGA1 in regulating MPP^+^-induced autophagic flux impairment. We and other previous studies (Lim et al., 2011; Miyara et al., 2016) demonstrated that mild MPP^+^ exposure predominantly inhibited impaired autophagosome degradation in MN9D cells (Supplementary Figure 3). Twenty-four hours after transfection with two specific HMGA1 siRNA or NC, MN9D cells were treated with 200 μM MPP^+^ for another 48 h with or without 100 nM Baf-A1 for the last 4 h and subjected to Western blot analysis. As expected, knockdown of HMGA1 further increased the accumulation of LC3-II induced by MPP^+^ treatment, while with the cotreatment of Baf-A1, HMGA1 silencing did not further increased the MPP^+^-induced LC3-II expression (Figures 4A,C). Moreover, levels of p62 were also higher in the MPP^+^-treated HMGA1 silencing cells compared with the MPP^+^-treated alone group (Figures 4A,C). To further support our hypothesis, the flux rate of autophagy was measured with an mRFP-GFP-LC3 reporter construct (Mizushima et al., 2010). We found that in control cells, most of the puncta were red only (autolysosomes, mRFP^+^/GFP^−^), confirming that autophagosomes are degraded by acidic lysosomes. While MPP^+^ treatment significantly increased the number of yellow puncta (autophagosomes, mRFP^+^/GFP^+^) and decreased the number of red-only puncta, indicating the partial blockage of autophagosome degradation. Moreover, knockdown of HMGA1 in the MPP^+^-treated cells led to more yellow puncta and less red-only puncta (Figures 4D,E). Altogether, these results provided direct evidence that HMGA1 depletion could accelerate cell death through exacerbating MPP^+^-induced autophagy impairment.

### HMGA1 Forms a Negative Feedback Loop With miR-103/107

**Figure 5:**
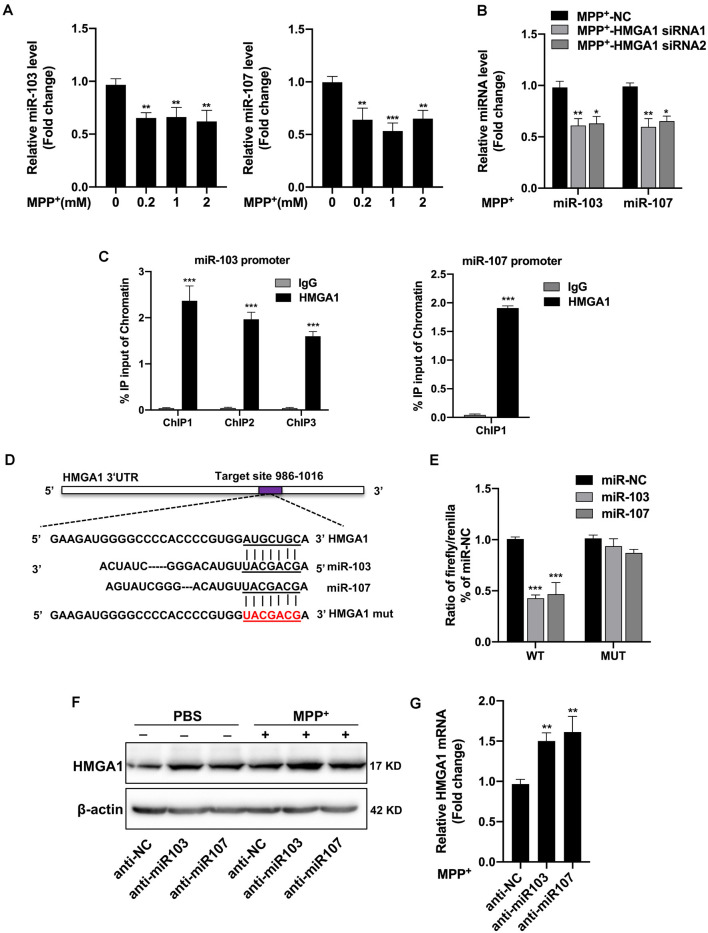
High-mobility group A1 (HMGA1) forms a negative feedback loop with miR-103/107 family. **(A)** MN9D cells were treated with different concentrations of MPP^+^ for 48 h, and expression levels of miR-103/107 family were analyzed by quantitative real-time PCR (qRT-PCR). **(B)** qRT-PCR analysis of miR-103/107 family in MN9D cells treated with MPP^+^ (200 μM) following transfection of cells with HMGA1 siRNA or NC. **(C)** Chromatin immunoprecipitation (ChIP) assays were carried out in MN9D cells. qRT-PCR was performed with specific primers for predicted promoter binding sites. **(D)** Illustration of putative miR-103/107 binding site in HMGA1. The potential complementary residues are underlined, and mutated bases are indicated in red. **(E)** Relative luciferase activity was measured in HEK293T cells at 48 h cotransfection of miR-103 or miR-107 mimics with Luc-HMGA1-WT or Luc-HMGA1-MUT vector. A representative experiment with triplicates of two independent experiments is shown. **(F,G)** MN9D cells were treated with miR-103/107 inhibitors or anti-NC and treated with 200 μM MPP^+^ or phosphate-buffered saline (PBS) for 24 h. **(F)** Western blot analysis and **(G)** qRT-PCR analysis were performed to detect HMGA1 protein and messenger RNA (mRNA) expression. Data are expressed as mean ± SEM (*n* = 3). *P < 0.05, **P < 0.01, and ***P < 0.001 vs. control using one-way ANOVA followed by Bonferroni test or two-tailed unpaired Student’s *t* test.

We then began to ask how HMGA1 regulates MPP^+^-mediated MN9D autophagy. It has been shown that HMGA1 is involved in the direct regulation of miRNAs expression (De Martino et al., 2009; Fu et al., 2018). Recently, an miRNA family, miR-103/107, has been reported to coordinately regulate autophagy and apoptosis (Park et al., 2016). They are both highly expressed in brain tissue and especially enriched in neuron (Wang et al., 2014). However, it is still unknown whether miR-103/107 participated in the HMGA1-mediated autophagy of MN9D cells. We than surveyed the miR-103/107 abundance in MN9D cells. It showed that both miR-103/107 were obviously decreased following different concentrations of MPP^+^ treatment (Figure 5A). Considering its direct regulation on miRNA expression, we speculated whether HMGA1 affected the expression of miR-103/107. Surprisingly, HMGA1 silencing by two specific siRNAs in MN9D cells resulted in further downregulation of miR-103/107 induced by MPP^+^ treatment (Figure 5B). To understand how miR-103/107 expression was regulated by HMGA1, we analyzed the promoter regions of miR-103 and miR-107 (2-kb upstream the transcription start site), respectively, and found the presence of putative HMGA1-binding sites by using ConTra v3 web server^1^ (Kreft et al., 2017). ChIP assay with anti-HMGA1 or rabbit IgG antibodies was then performed. The results shown in Figure 5C demonstrated that HMGA1 was able to bind to these sequences in MN9D cells. These results suggest that HMGA1 can directly regulate the transcription of the miR-103/107 by binding to their promoters, and elevated HMGA1 can sustain their expression in MPP^+^-damaged MN9D cells.

Moreover, through bioinformatics prediction using starBase v2.0^2^, we surprisingly found that HMGA1 presented putative binding site for miR-103/107 family. Therefore, we constructed luciferase vectors of wild HMGA1 3′-UTR (Luc-HMGA1-WT) and the mutated form (Luc-HMGA1-MUT). Dual-luciferase assays revealed that both miR-103/107 greatly reduced the luciferase activities of Luc-HMGA1-WT in HEK293 cells, while it had no effect on the mutated form of HMGA1 (Figures 5D,E). Furthermore, to validate the influence of miR-103/107 on HMGA1 expression, MN9D cells were transfected with miR-103/107 inhibitors and treated with 200 μM MPP^+^ or PBS for 24 h. As expected, the knockdown of miR-103/107 resulted in further increased expression of HMGA1 both in protein (Figure 5F) and mRNA levels (Figure 5G). Taken together, we show evidences that HMGA1, together with the miR-103/107 family, forms a negative feedback loop during the progress of MPP^+^-induced cell death.

### miR-103/107 Regulate MPP^+^-Induced Autophagy by Targeting CDK5R1/CDK5 Signaling

**Figure 6:**
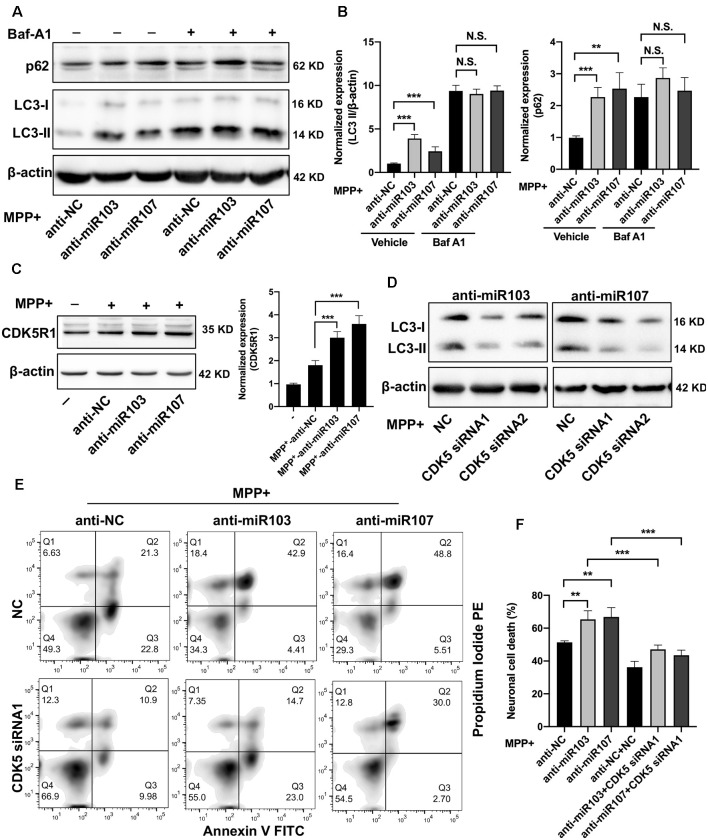
MiR-103/107-CDK5R/CDK5 signaling regulate MPP^+^-induced autophagy impairment. **(A,B)** MN9D cells were treated with miR-103/107 inhibitors or anti-NC and treated with 200 μM MPP^+^ or phosphate-buffered saline (PBS) for 48 h in the presence or absence of Baf A1 (100 nM) for the last 4 h. **(A)** Immunoblot analysis of LC3 and p62 levels and **(B)** quantification were shown. **(C)** Immunoblot analysis of CDK5R1 in MN9D cells treated with MPP^+^ (200 μM) following transfection of cells with miR-103/107 inhibitors or anti-NC. (**D–F**) MN9D cells were cotransfected with miR-103/107 inhibitors in the presence of CDK5-specific siRNAs, followed by the treatment of 200 μM MPP^+^ for 48 h. Cells were harvested and assessed for **(D)** LC3 expression and cell death. Representative images of MN9D cell deaths assessed by **(E)** flow cytometry analysis and **(F)** quantification were shown. Data are expressed as mean ± SEM (*n* = 3). N.S., not statistically significant, *P < 0.05, **P < 0.01, and ***P < 0.001 vs. control using one-way ANOVA followed by Bonferroni test.

We next validated the role of miR-103/107 family in the regulation of autophagy in MN9D cells. Twenty-four hours after transfection with miR-103/107 inhibitors or control, MN9D cells were treated with 200 μM MPP^+^ for another 48 h with or without 100 nM Baf-A1 for the last 4 h. In line with a previous report (Park et al., 2016), the loss of miR-103/107 further increased the amount of LC3-II induced by MPP^+^ treatment, while in the presence of Baf-A1, no obvious difference was detected (Figures 6A,B). Moreover, in MN9D cells treated with miR-103/107 inhibitors, an increase in p62 levels was also noted compared with control treatment (Figures 6A,B). In contrast, ectopic overexpression of miR-103/107 alleviated the increased amount of LC3-II and p62 induced by MPP^+^ treatment (Supplementary Figure 4). These results provided strong evidence that the autophagy flux inhibition induced by MPP^+^ was mediated, at least partially, by miR-103/107 family. Accordingly, flow cytometry analysis showed that anti-miR-103/107 exposure led to higher cell death compared with anti-NC treatment (Figures 6C,F). Considering that cyclin-dependent kinase 5 (CDK5) has been reported to be an essential mediator for autophagy and neuronal loss in models of PD (Wong et al., 2011; Cheung and Ip, 2012), and its activatory subunit CDK5R1 (also known as p25 or p35) is a well-known target of miR-103/107 (Moncini et al., 2011, 2017), we tended to determine whether CDK5R1/CDK5 signaling mediates the effect of miR-103/107 family. Indeed, anti-miR-103/107 treatment of MN9D cells increased the levels of CDK5R1 (Figure 6C), and knocked down endogenous CDK5 markedly attenuated LC3-II accumulation (Figure 6D, Supplementary Figure 5) and neuronal cell death resulting from miR-103/107 inhibition in response to MPP^+^ treatment (Figures 6E,F). Collectively, these findings strongly indicate the involvement of CDK5R1/CDK5 signaling pathway in miR-103/107-mediated autophagy and neuronal cell death.

### HMGA1 Regulates Dopamine Cell Death Through miR-103/107/CDK5 Signaling

**Figure 7:**
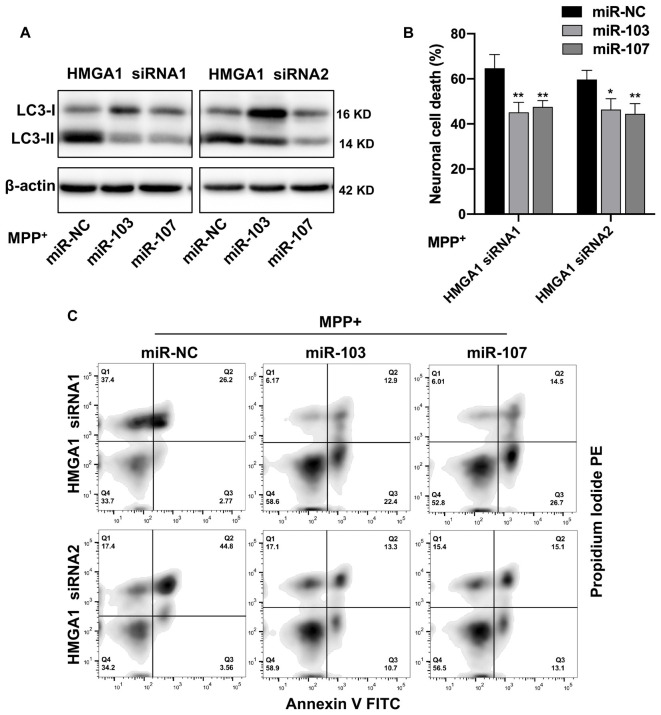
miR-103/107/CDK5 signaling mediates high-mobility group A1 (HMGA1)-regulated dopamine cell death. **(A)** MN9D cells were cotransfected with HMGA1 small-interfering RNAs (siRNAs) in the presence of miR-103/107 mimics or miR-NC, followed by the treatment of 200 μM MPP^+^ for 48 h. Cells were harvested and assessed for LC3 expression and cell death. **(B,C)** Representative images of MN9D cell deaths assessed by **(C)** flow cytometry analysis and **(B)** quantification were shown. Data are expressed as mean ± SEM (*n* = 3). *P < 0.05, **P < 0.01 vs. control using one-way ANOVA followed by Bonferroni test.

We next evaluated whether miR-103/107 signaling pathway participated in the HMGA1-mediated autophagy and cell death of MN9D cells. We knocked down endogenous HMGA1 and ectopic overexpressed miR-103/107 with miRNA mimics simultaneously and found that the elevation of LC3-II (Figure 7A), as well as neural cell death (Figures 7B,C), induced by HMGA1-deleption, was abrogated in MPP^+^-treated MN9D cells in the presence of miR-103/107 mimics. Taken together, our observations reveal that loss of HMGA1 aggravated autophagy-mediated neuronal loss in MPP^+^-treated MN9D cells, which is dependent on miR-103/107/CDK5 signaling pathway.

### HMGA1-Mediated Loop Contributes to the Death of TH^+^ Neurons in the Subacute MPTP Mouse Model of PD

**Figure 8:**
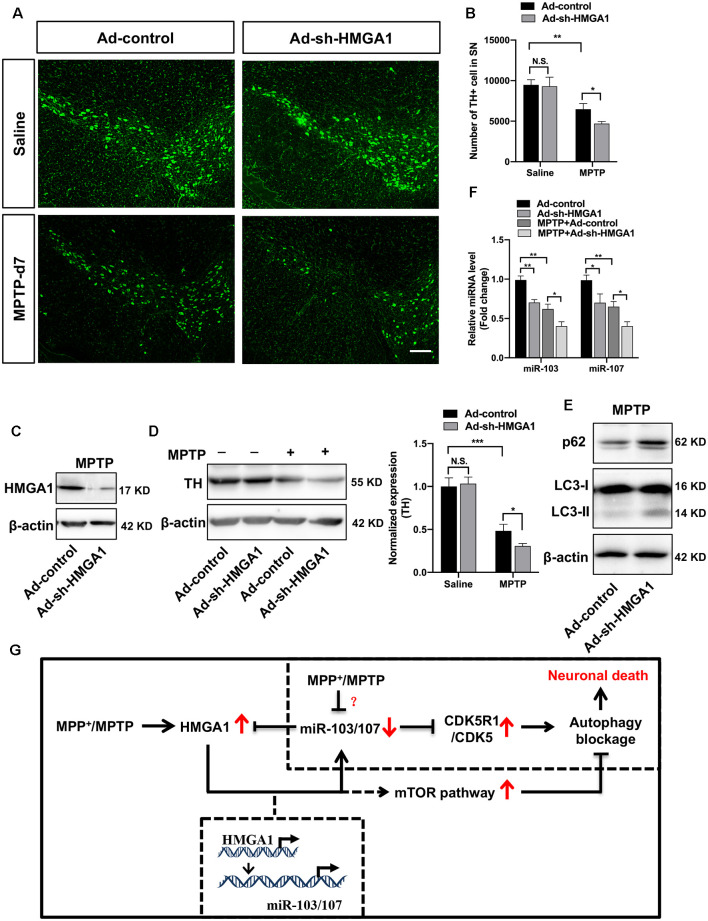
High-mobility group A1 (HMGA1)-mediated loop contributes to the death of TH^+^ neurons in the subacute 1-methyl-4-phenyl-1,2,3,6-tetrahydropyridine (MPTP) mouse model of Parkinson’s disease (PD). Ad-sh-HMGA1 or Ad-control were stereotaxically delivered into the right substantia nigra region of C57BL/6 mice as described in “Materials and Methods” section. Four weeks later, MPTP-HCl (25 mg/kg free base) or equivalent saline was administered subcutaneously to the mice daily for five consecutive days. **(A)** Representative immunofluorescence staining for TH (green) in the substantia nigra region 7 days after the last MPTP injection. Scale bar: 100 μm. **(B)** Stereological counting of TH-positive cells in the entire substantia nigra are shown (*n* = 4 mice per group). **(C,F)** Total protein and RNA isolated from dissected ventral mesencephalon were subjected to Western blot analysis of **(C)** HMGA1 levels, **(D)** TH levels, **(E)** LC3 and p62 levels, and qRT-PCR analysis of **(F)** miR-103/107 family levels. **(G)** Schematic illustration of the proposed model. MPP^+^ treatment inhibits autophagy flux through downregulating miR-103/107 and activating CDKR51/CDK5 signaling pathway. While MPP^+^/MPTP-induced HMGA1 elevation counterparts the effect of miR-103/107 downregulation by directly binding to their promoters, respectively, sustaining their expression in MPP^+^-damaged MN9D cells. Moreover, HMGA1 is also a direct target of miR-103/107. Thus, HMGA1 forms a negative feedback loop with miR-103/107 and drives neuroprotection through autophagy modulation. In addition, HMGA1 may modulate autophagosome formation through the activity of the mTOR pathway. Data are expressed as mean ± SEM (*n* = 6 mice per group). N.S., not statistically significant. *P < 0.05, **P < 0.01 vs. control using one-way ANOVA followed by Bonferroni test. ***P < 0.001.

To further demonstrated the physiological roles of HMGA1 on neural cell death *in vivo*, the well-characterized subacute MPTP mouse model of PD was used. As shown in Figures 2A–C, MPTP treatment induced the expression of HMGA1, while it suppressed the expression of miR-103/107 (Figure 8F) in the SN tissue, indicating their important role in regulating MPTP-induced neurotoxicity. Adenovirus encoding shRNA against HMGA1 (Ad-sh-HMGA1) or control shRNA (Ad-control) were stereotaxically injected into the right SN region. Western blot analysis showed that Ad-sh-HMGA1 efficiently reduced HMGA1 in the SN region (Figure 8C). Four weeks later, mice were exposed to the subacute MPTP paradigm. TH+ immunofluorescence staining 7 days after the last MPTP injection revealed that, while HMGA1 depletion alone *in vivo* did not affect the nigrostriatal dopaminergic system, it augmented MPTP-induced TH+ neurons death in the SN region (Figures 8A,B). Western blotting of nigral tissue lysates showed that, in Ad-control injected mice, MPTP injections significantly reduced TH expression, whereas in Ad-sh-HMGA1-injected mice, HMGA1 depletion greatly enhanced MPTP-induced TH loss (Figure 8D). Moreover, LC3-II, p62, and miR-103/107 expression changes were also detected in the SN tissues (Figures 8E,F). Similar results were obtained *in vivo* compared with the results obtained from MN9D cells. In conclusion, our results further support that blocking endogenous HMGA1-mediated loop aggravates TH+ neurons degeneration in the MPTP model of PD.

## Discussion

Even though the pathogenesis of Parkinson’s disease is still not fully decoded, accumulating evidence has linked autophagy dysfunction to a number of neurodegenerative disorders including PD (Nixon, 2013; Menzies et al., 2017). Indeed, autophagy deregulation have been detected in the SN of PD patients and *in vivo* experimental animal models (Wang et al., 2016). PD-related genes, for example, SNCA (Winslow and Rubinsztein, 2011), PINK1/Parkin (Vincow et al., 2013), KRRK2 (Orenstein et al., 2013), and so on, are known to be involved in the regulation of autophagic pathway. However, there has been a long-standing controversy concerning the precise nature of autophagy in neurodegenerative diseases during the past decades. Recent findings suggest that either excessive autophagic activation (Wong et al., 2011) or basal neuronal autophagy defect eventually leads to autophagic programmed cell death (Hara et al., 2006; Komatsu et al., 2006). It is true that basal neuronal autophagy is required for clearance of ubiquitinated proteins and is essential for neuron survival. While aberrant activation of autophagy may also result in neuronal loss. There are other studies demonstrate autophagic flux impairment due to blocking autophagosome degradation (Dehay et al., 2010; Lim et al., 2011). Thus, knowledge of how the critical autophagic events happen during neurodegeneration and their association with continuous neuron damage is of great importance. In the present study, we uncover a novel neuroprotective role for HMGA1 through functioning as an essential autophagy regulator in both cell culture and murine models of PD (Figure 8G).

HMGA1 is a well-known architectural factor that frequently elevates in rapidly proliferative cells (such as embryonic stem cells) and diverse cancer cells (Sumter et al., 2016), with absent or low levels in normal, differentiated, adult tissues (Giancotti et al., 1993). Accordingly, it has been shown that high levels of HMGA1 expression play a central role in driving cancer progression and aggressiveness (Shah and Resar, 2012). In addition, HMGA1 was also found to be overexpressed in intestinal stem cells and exhibited a reveal a pivotal role in stem cell self-renewal (Xian et al., 2017). These studies demonstrate that HMGA1 appears to exert diverse functions in multiple diseases. However, its role in neurodegeneration diseases, such as PD, is remain elusive. Here, we focused on the novel role of HMGA1 in regulating neural cell death in PD, which has not yet been clearly illustrated. Indeed, we demonstrated for the first time that HMGA1 was rapidly induced following neuronal injury in MN9D cell culture, supporting the involvement of HMGA1 in PD pathogenesis. HMGA1 has been shown to contribute to carcinogenesis by inactivating apoptosis signal pathway (Cheng et al., 2019). We showed that inhibition of endogenous HMGA1 further promoted MPP^+^-induced neural cell death, as confirmed by the flow cytometry, indicating a novel potential neuroprotective role for HMGA1 in dopaminergic cells during neuronal cell death. Moreover, we found that HMGA1 knockdown alone greatly induced the accumulation of endogenous LC3-II and p62, demonstrating a basal autophagic flux impairment of MN9D cells resulting from partial autophagosome degradation blockage, which further exacerbated MPP^+^-induced autophagy impairment and promoted neural cell death. Our observation provided direct evidence that HMGA1 depletion could accelerate cell death through exacerbating MPP^+^-induced autophagy impairment. Notably, previous studies demonstrated that HMGA1 knockdown increased autophagosome formation by constraining the activity of the mTOR pathway in cancer cells (Conte et al., 2017). They further showed that such an increase in autophagosomes formation is not mirrored by an equal increase in their maturation, with a consequent reduction in the autophagic flux. We also observed the activation of mTOR-dependent autophagic pathway in HMGA1-knockdown MN9D cells (Supplementary Figure 2). However, we could not observe any significant increase in LC3-II with the cotreatment of Baf-A1, indicating that HMGA1 knockdown decreases autophagic flux mainly by partially blocking autophagosome degradation in MN9D cells, which was further supported by the measurement of flux rate of autophagy using mRFP-GFP-LC3 construct. In fact, an earlier study has suggested that HMGA1 function depends on the specific cellular context (Martinez Hoyos et al., 2004).

We further extended our *in vitro* findings to *in vivo* experimental models of PD. In agreement with our *in vitro* data, we observed the upregulation of HMGA1 after the last MPTP injection, reaching a maximum by 2–4 days. Moreover, we also found that ablation of HMGA1 in SN region significantly induced more TH+ neurons death with the treatment of MPTP, accompanied with the elevation of LC3-II and p62. Thus, considering the autophagosome accumulation in PD resulting from defective lysosomal system (Dehay et al., 2010), it may be beneficial for HMGA1 elevation in protecting dopaminergic neural cells through maintaining proper end-stage autophagy.

To uncover the detailed mechanism, we showed that HMGA1 forms a negative feedback loop with miR-103/107-CDK5 signaling pathway to regulate the MPTP/MPP^+^-induced autophagy impairment and neural cell death (Figure 8G). Our results demonstrated that HMGA1 could enhance the transcription of miR-103/107 family by directly targeting their promoters, consistent with its particular role in the regulation of miRNAs expression.

We found that MPTP/MPP^+^ treatment significantly decreased the expression of miR-103/107 family both *in vitro* and *in vivo* model of PD. Therefore, the elevated expression of HMGA1 in PD would sustain the levels of miR-103/107, preventing their continuous decrease in PD. Obviously, there are other stronger negative regulators indued by MPTP/MPP^+^ treatment finally decreasing the expression of miR-103/107 family. Furthermore, the specific role of miR-103/107 family during the progression of PD is still unclear. Previous studies have shown the important role of miR-103/107 family in regulating autophagy, especially end-stage autophagy, mediated by CDK5R1/CDK5 signaling pathway (Wang et al., 2018, 2020). In addition, CDK5 has been demonstrated to induce MPTP-induced neuronal death by regulating autophagy (Wong et al., 2011) and survival factor MEF2, antioxidant enzyme Prx2, and DNA damage repair enzyme Ape1 (Cheung and Ip, 2012). Thus, it is plausible that the effect of miR-103/107 family on autophagy contributes to the pathophysiology of PD. Indeed, we observed that loss of miR-103/107 further augmented the MPP^+^-induced autophagosome degradation partial blockage and neural cell death by targeting CDK5R1/CDK5 signaling pathway. Ectopic overexpressed miR-103/107 simultaneously could abrogate the effect of endogenous HMGA1 knockdown, indicating that miR-103/107 family mediated the HMGA1-induced autophagy and neuronal death. Finally, concerning the transcriptional mechanisms of HMGA1 upregulation in MN9D cells, we found that HMGA1 was also the direct target of miR-103/107 family. Loss of miR-103/107 resulted in further increased expression of HMGA1 both in protein and mRNA levels. Therefore, we conclude that HMGA1, together with miR-103/107 family, forms a negative feedback loop during the progression of autophagy and neural cell death in PD.

In summary, our study provided evidence depicting a pivotal role for HMGA1 in the regulation of neuronal loss in PD models through autophagy regulation. We identify a regulatory circuit, which is composed of HMGA1, miR-103/107 family, and CDK5R1/CDK5 signaling pathway, mediating a regulatory signal that is critical for maintaining autophagic flux balance and preventing neuronal death in PD. Future studies are required to fully clarify the role of HMGA1 in PD pathogenesis and the development of autophagy regulators as therapeutics for PD.

## Data Availability Statement

The original contributions presented in the study are included in the article/Supplementary Material, further inquiries can be directed to the corresponding author/s.

## Ethics Statement

The animal study was reviewed and approved by Southern Medical University Ethics Committee.

## Author Contributions

SZ, GL, and YY conceived, designed, and guided this study. YL contributed to the development of this study. GL, WL, and BW performed cellular experiments and prepared Figures 1–7. CQ and YY conducted animal experiments and prepared Figure 8. GL, WL, and YY wrote the manuscript. All authors contributed to the article and approved the submitted version.

## Conflict of Interest

The authors declare that the research was conducted in the absence of any commercial or financial relationships that could be construed as a potential conflict of interest.

## Footnotes

^1^http://bioit2.irc.ugent.be/contra/v3

^2^http://starbase.sysu.edu.cn/
