# Supplementary material for: HMGA1 Induction of miR-103/107 Forms a Negative Feedback Loop to Regulate Autophagy in MPTP Model of Parkinson’s Disease
Source: Front Cell Neurosci. 2021 Jan 18;14:620020. doi: 10.3389/fncel.2020.620020 (PMC7847849; doi:10.3389/fncel.2020.620020)
Supplement: Supplementary file 1 [file Data_Sheet_1.PDF]

Supplementary Material

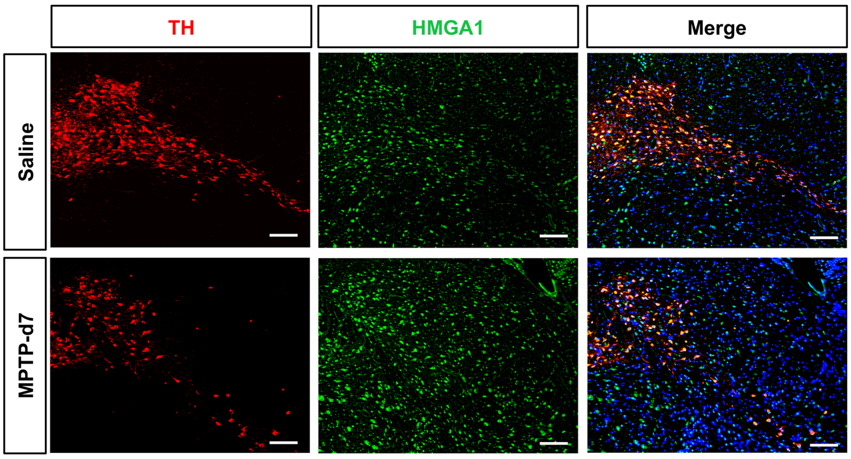

Supplementary Figure 1. HMGA1 is highly induced in nigral DA neurons in MPTP mouse model of PD

Representative immunofluorescence double staining for TH (red) and HMGA1 (green) showing increased HMGA1 expression in nigral dopaminergic neurons. Scale bar: 100 μm.

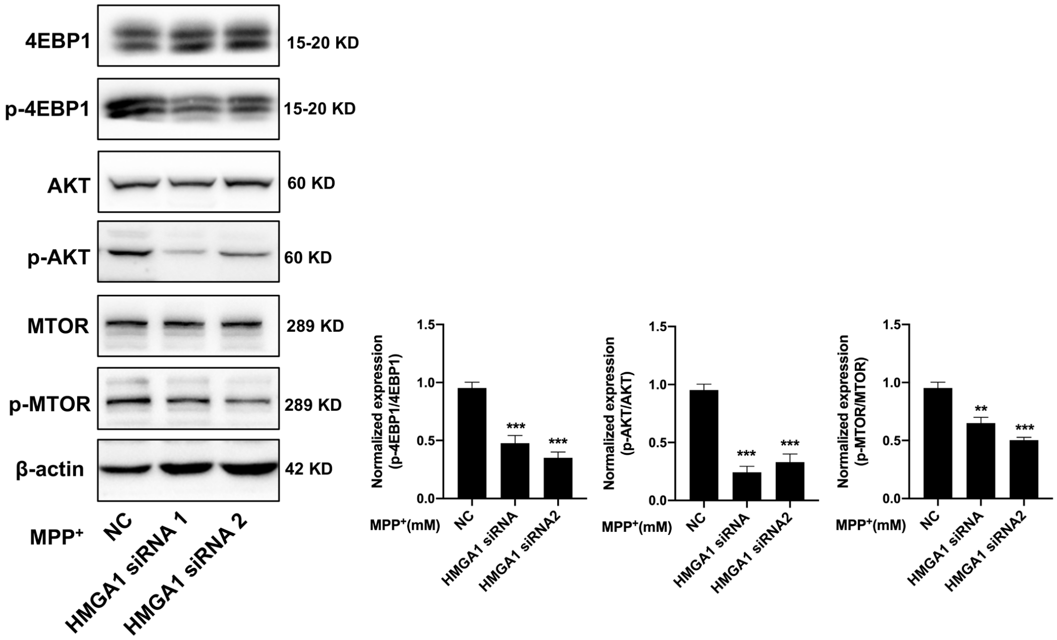

Supplementary Figure 2. HMGA1 knock down activates mTOR-dependent autophagic pathway

MN9D cells were transfected with two HMGA1 siRNAs or NC, respectively, and exposed to MPP$^+$ (200 μM) for 48h. Cells were harvested and assessed for mTOR, p-mTOR, 4EBP1, p-4EBP1, AKT and p-AKT expression.

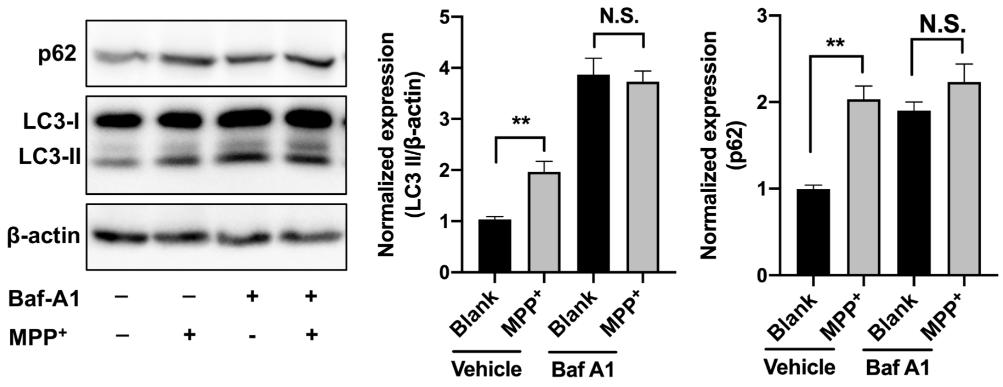

Supplementary Figure 3. MPP$^+$ induced autophagy impairment in MN9D cells
MN9D cells were treated with 200 μM MPP$^+$ for 48h in the presence or absence of lysosomal pump inhibitor Bafilomycin A1 (100nM) for the last 4h. Immunoblot analysis of LC3 and p62 levels were shown.

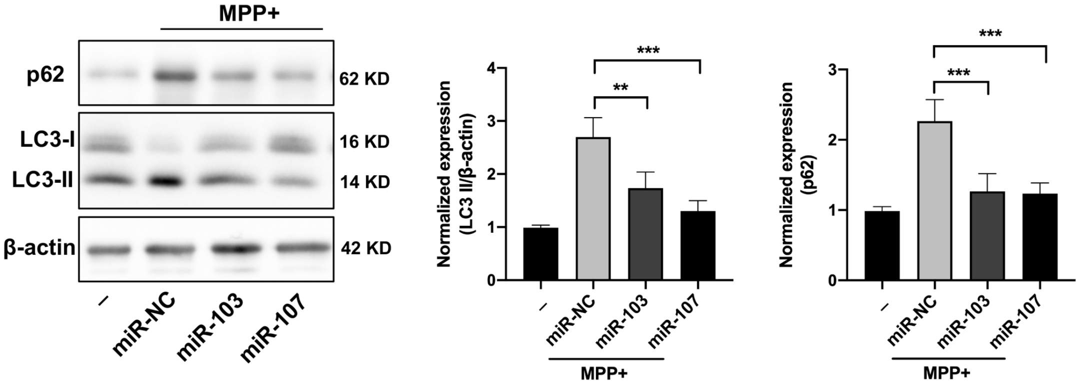

Supplementary Figure 4. Overexpression of miR-103/107 alleviates MPP$^+$ induced autophagy impairment
MN9D cells were treated with miR-103/107 mimics or miR-NC and treated with 200 μM MPP$^+$ or PBS for 48h in the presence or absence of Baf A1 (100nM) for the last 4h. Immunoblot analysis of LC3 and p62 levels and quantification were shown.

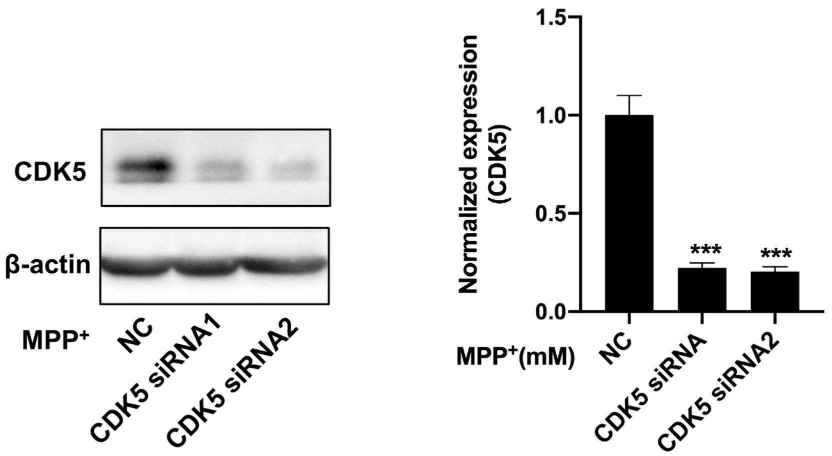

Supplementary Figure 5. Effectivity of CDK5 siRNAs

MN9D cells were transfected with CDK5 siRNA or NC, and CDK5 levels were analysed by western blot.
